# Metabolic Fingerprinting with Fourier-Transform Infrared (FTIR) Spectroscopy: Towards a High-Throughput Screening Assay for Antibiotic Discovery and Mechanism-of-Action Elucidation

**DOI:** 10.3390/metabo10040145

**Published:** 2020-04-09

**Authors:** Bernardo Ribeiro da Cunha, Luís P. Fonseca, Cecília R.C. Calado

**Affiliations:** 1 iBB—Institute of Bioengineering and Biosciences, Instituto Superior Técnico (IST), Universidade de Lisboa (UL), Av. Rovisco Pais, 1049-001 Lisboa, Portugal; luis.fonseca@tecnico.ulisboa.pt; 2 ISEL—Instituto Superior de Engenharia de Lisboa, Instituto Politécnico de Lisboa (IPL), R. Conselheiro Emídio Navarro 1, 1959-007 Lisboa Portugal; ccalado@deq.isel.pt

**Keywords:** antibiotic discovery, chemometrics, *Escherichia coli*, Fourier-Transform infrared (FTIR) spectroscopy, high-throughput screening, mechanism-of-action (MOA), metabolic fingerprinting, multivariate analysis

## Abstract

The discovery of antibiotics has been slowing to a halt. Phenotypic screening is once again at the forefront of antibiotic discovery, yet Mechanism-Of-Action (MOA) identification is still a major bottleneck. As such, methods capable of MOA elucidation coupled with the high-throughput screening of whole cells are required now more than ever, for which Fourier-Transform Infrared (FTIR) spectroscopy is a promising metabolic fingerprinting technique. A high-throughput whole-cell FTIR spectroscopy-based bioassay was developed to reveal the metabolic fingerprint induced by 15 antibiotics on the *Escherichia coli* metabolism. Cells were briefly exposed to four times the minimum inhibitory concentration and spectra were quickly acquired in the high-throughput mode. After preprocessing optimization, a partial least squares discriminant analysis and principal component analysis were conducted. The metabolic fingerprints obtained with FTIR spectroscopy were sufficiently specific to allow a clear distinction between different antibiotics, across three independent cultures, with either analysis algorithm. These fingerprints were coherent with the known MOA of all the antibiotics tested, which include examples that target the protein, DNA, RNA, and cell wall biosynthesis. Because FTIR spectroscopy acquires a holistic fingerprint of the effect of antibiotics on the cellular metabolism, it holds great potential to be used for high-throughput screening in antibiotic discovery and possibly towards a better understanding of the MOA of current antibiotics.

## 1. Introduction

The discovery of antibiotics has been considered a miracle of modern medicine, but since the golden age of antibiotic discovery, when most classes were introduced, innovation has been slowing to a halt [1]. The genomics era inspired target-based screening but hits generally proved ineffective at reaching their target within the cell and not a single new drug reached the market from target-based screening programs [2]. Nowadays, phenotypic screening is a preferred strategy for antibiotic discovery, mostly because compounds that are effective against whole cells have a higher likelihood of becoming candidate molecules and can target poorly understood metabolic pathways [3]. However, these assays do not reveal the Mechanism-Of-Action (MOA) of candidate compounds, which requires considerable efforts at a later stage of the discovery process [4]. This results in higher rates of rediscovery, low probabilities of finding compounds with unique biological and/or chemical properties and limited insight in the pharmacological target. Additionally, phenotypic screening does not explore the chemical grey matter, i.e., compounds capable of inducing some level of phenotypic modulation, but without sufficient potency to induce cell death or growth inhibition, which can be a source of compounds suitable for lead optimization with medicinal chemistry techniques [5,6]. Antibiotic discovery is a very challenging task, but identifying the MOA has proven equally challenging [7]. Currently, determining the MOA of antibiotics is still a bottleneck of the phenotypic screening discovery process, for which metabolomics holds great potential. As such, the ability to rapidly infer MOA and, if possible, the biomolecular target of antibiotics is increasingly important given the pressing need for new antibiotics. Currently, screening hundreds of thousands of compounds is a reasonable throughput of a drug discovery program, in part due to the ease in synthetizing bioactive compounds, and in part given the increasing availability of natural product libraries [8]. Two concepts are relevant when discussing MOA identification. One is determining the molecular pathways affected by a given compound:the drug effects.The second is the specific compound—substrate interactions:the drug target [9]. Although both concepts are very important in antibiotic discovery, given the exploratory purpose of this study, a less stringent definition of MOA identification was used. Herein, we refer to MOA elucidation as an approximation using a mechanism-specific fingerprint, rather than the identification of the specific targets of a given molecule, and the pathways affected, which formally constitute MOA identification.

Conventional MOA studies are based on macromolecular synthesis assays; however, these assays are typically slow, laborious, low resolution, low accuracy, and low throughput [10]. An equally limited alternative lies in biochemical approaches, for instance the use of affinity chromatography to identify the exact biomolecule to which a candidate molecule binds [11,12]. However, this is somewhat a fishing expedition in the sense that it requires the happy combination of a high-affinity small molecule with a fairly abundant protein receptor [13]. Another important issue of conventional MOA assays is that a large quantity of the test compound is required, which is not always attainable. Recently, genome-wide transcriptional or translational profiles have been used to reveal the target of candidate molecules, but, more often than not, these profiles overwhelmingly reflect indirect stress responses rather than the specific sequence of events that results in the inhibitory effect [14]. Since the metabolome is at the lower end of the Omics cascade, it reflects the substrates and products of various metabolic enzymes, and thereby can be used to pinpoint drug-induced inhibition. As such, early metabolomics studies into the MOA of antibiotics looked at shifts of metabolite concentrations, induced by a single molecule, to identify its specific target(s) [7,15]. As the understanding of MOA grew more complex, from targets to networks, metabolomics has been increasingly used to build comprehensive multi-parametric profiles of the MOA [16]. These profiles provide a genome-scale characterization of the drug-induced effects, which extends beyond non-metabolic targets [17]. One advantage of metabolic profiling is that it considers both on-target and off-target effects, which together produce the antibiotic effect, via an underlying MOA, of a molecule. Therefore, metabolomics studies on MOA focused on obtaining metabolic profiles. The comparative metabolic profiling of a pair of isogenic methicillin-susceptible and -resistant *Staphylococcus aureus* has emphasized the metabolic alterations that are specific to the MOA of three antibiotics acting on the major biosynthetic pathways, i.e., cell wall, DNA, and protein biosynthesis [18]. Additionally, the metabolic profiling of drug exposure has also been used, together with metabolic and chemogenomic profiles of single-deletion strains, to predict epistatic drug interactions. This enables the rational design of drug combinations by identifying nonantibiotic compounds that, when combined, have antimicrobial activity [19]. Metabolic profiling also plays a role in the dereplication and guided fractioning of novel natural products with antimicrobial properties [20]. 

Regarding studies dedicated towards high-throughput MOA elucidation, nuclear magnetic resonance has the advantage of being faster and less expensive, albeit only capable of identifying highly abundant metabolites. One approach to tackle this has been to analyze both the intracellular fingerprints and extracellular footprints, which resulted in more comprehensive and specific metabolic profiles [21]. Another approach relies on Mass Spectrometry (MS), which can be used to identify a wider range of metabolites with higher sensitivity, especially when combined with chromatographic separation techniques. Until recently, MS-based metabolomics could only be applied to study the MOA of individual molecules, but sufficient throughput can be attained with an untargeted approach, where thousands of ion peaks are detected from individual samples, although annotation is still the bottleneck, i.e., identifying metabolites from said peaks [22]. In fact, untargeted metabolomics can achieve the required throughput to systematically ascertain the MOA of moderately large collections of antibiotics [23]. However, to reach the 10–100x increase in throughput required for large-scale studies, there has to be a compromise of either coverage and/or separation, which currently requires dismissing the chromatographic step [24]. As such, by sacrificing resolution, high-throughput untargeted metabolomics using flow injection electrospray has predicted the MOA of uncharacterized antimycobacterial compounds from an industry-scale chemical library [25]. Within said compromise of resolution in favor of throughput, other analytical techniques could be better suited for the rapid handling of a large number of samples, with minimal preparation and manipulation, though they inherently yield less informative data. 

Fourier-Transform Infrared (FTIR) spectroscopy is an established metabolic fingerprinting technique particularly well suited for high throughput, which requires minimal sample handling, and is reagent-free and label-free [26]. Moreover, FTIR spectroscopy as a metabolic fingerprinting technique provides relevant chemical information to rapidly and reproducibly discern prominent changes in the metabolome [27], particularly those imposed by stress agents and antibiotics [28]. Additionally, FTIR microscopy has been successfully used to identify antibiotic resistance from clinical isolates [29]. FTIRS is especially promising because its characteristics bridge the gap between the low-throughput/high-information metabolomic assays and the high-throughput/low-information nature of phenotypic screening assays. While FTIRS does not result in comprehensive data on the metabolite level, the volume of biological information it yields allows for an enhanced assessment of the biomolecular phenomena underlying the antibiotic effect, which has been shown to be suitable in MOA-centric studies. In fact, some studies have dwelled into the ability of FTIRS in detecting antibiotic-specific fingerprints [29,30,31,32]. However, these studies either did not explore the full potential of high-throughput or were limited to a small number of antibiotics with considerably distinct MOA. Thus, it remains unclear whether FTIRS can be used to rapidly obtain metabolic profiles with sufficient sensitivity to distinguish very similar MOA, e.g., antibiotics acting on the same major biosynthetic pathway, and within, antibiotics of the same class.

The aim of this study was to explore FTIRS as a metabolic fingerprinting technique towards the high-throughput determination of antibiotics’ MOA. The fingerprint induced by 15 antibiotics on the *Escherichia coli* metabolism was sufficiently specific to allow for a clear distinction between the different antibiotics across three independent cultures, conducted on different days. The range of antibiotics tested includes nine classes acting on key biosynthetic pathways: protein, DNA, RNA, and cell wall synthesis. *E. coli* cells were exposed to the antibiotics at four times the minimum inhibitory concentration for three hours, which ensured an inactivation of at least 90% across all samples. Spectra were preprocessed with the Savitzky–Golay (SG) derivative filter followed by Loopy Multiplicative Scatter Correction (LMSC). After both SG and LMSC parameters were optimized, the dataset was analyzed with Partial Least Squares Discriminant Analysis (PLS-DA) and Principal Component Analysis (PCA) so as to consolidate the PLS-DA predictions with an unsupervised algorithm. Adequate separation between the MOA at the level of major biosynthetic pathway affected was obtained independently of the analysis algorithm, and this separation extended to the level of antibiotic-specific fingerprints, which is a positive indication that FTIRS is suited to the elucidation of antibiotics MOA. Moreover, all samples exposed to an antibiotic were clearly plotted separately from the controls, which is important to identify novel molecules with an antibiotic effect in the context of a screening assay towards antibiotic discovery. 

## 2. Results and Discussion

### 2.1. Minimum Inhibitory Concentrations (MICs) and Bacterial Inactivation for FTIR Readings

To evaluate FTIR spectroscopy as a metabolic fingerprinting technique suitable for distinguishing the MOA of different antibiotics, *E. coli* was exposed to 15 compounds belonging to different classes and acting on different key biosynthetic pathways (Table 1). The MIC of each compound was determined using standard methods, and these have been reported (Table 1). Additionally, the average inactivation of independent cultures exposed to antibiotics prior to FTIR readings was calculated (Table 1). This was done to verify that four times the MIC was sufficient to obtain a cellular inactivation of over 90% for FTIR measurements. This was particularly important because antibiotic exposure for FTIR measurements was conducted at a cell density three orders of magnitude higher than MIC testing, which was required to obtain sufficient biomass for spectra acquisition. Although the relationship between inoculum size and antimicrobial activity is not linear [33,34], previous studies have shown that four times the MIC is a suitable concentration for metabolomics analysis of antibiotics MOA [23]. Moreover, determining the average inactivation also attested that using a relative antibiotic concentration, e.g., four times the MIC, over an absolute concentration, e.g., 500 ug/mL, resulted in an equivalent antibiotic effect (i.e., equivalent inactivation) between different metabolic fingerprints. For instance, in the case of antibiotics that have both a bacteriostatic and bactericidal action, a rule of thumb is that bacteriostatic activity is determined by a ratio of the minimum bactericidal concentration to MIC above four [35]. Exposing *E. coli* to four times the MIC should therefore favor bactericidal activity, which is confirmed by the large inactivation reported. Importantly, this consolidates the notion that any spectral differences observed are most likely due to the specificity of the metabolic adaptations induced by each antibiotic, rather than dose-dependent variations. 

Furthermore, a 3-h time point was chosen to avoid unspecific stress responses generically triggered by antibiotic exposure and to ensure the absence of growth recovery after antibiotic exposure, as suggested [23]. Unspecific responses are elicited almost immediately, and any antibiotic-specific fingerprints typically become more evident as the exposure duration increases. In fact, a 30-min exposure at a concentration that minimizes cell death and lysis revealed a common metabolic response among bactericidal antibiotics, which evolved to antibiotic-specific metabolic responses at 60 min and more so at 90 min [36]. Increasing the antibiotic concentration well above the MIC seemingly accelerates antibiotic-specific metabolic adaptations, and fingerprints obtained after 30 min of exposure accurately reflect the MOA [21]. Other approaches combine the data from multiple timepoints [25], but this implies that more analysis have to be made for the same number of samples, and thereby reduces the overall throughput, hence it is preferably avoided. 

To prevent spectra reflecting any metabolic alterations induced by the antibiotic solvent, all antibiotics were dissolved in water, as conducted by others [21]. However, this route could result in undesirable effects, e.g., loss of potency, which had to be ruled out by determining the inactivation and MIC. Alternatively, dimethyl sulfoxide (DMSO) is used as a universal solvent, given its ability to dissolve both organic and inorganic compounds, along with its low toxicity. However, DMSO inhibits the rapid killing of diverse classes of antibiotics, even at concentrations as low as 1%. In fact, DMSO has been suggested to interfere with antibiotic lethality that is mediated by Reactive Oxygen Species (ROS), in a concentration and exposure duration dependent manner. As such, this protective effect is not constant across antibiotic classes, which can complicate MOA elucidation. Furthermore, this effect is not reflected on MIC values, as there are mechanistic differences between transient ROS-mediated killing and MICs. On the other hand, DMSO can alter cell membrane permeability, which is speculated to explain its inhibitory effect, and can result in an apparent increase in potency, as the entry into the cell by certain antibiotics is facilitated. Ultimately, these findings discourage the use of DMSO as a solvent for antimicrobials, especially in rapid-killing assays [37].

### 2.2. FTIR Preprocessing Optimization

**Figure 1:**
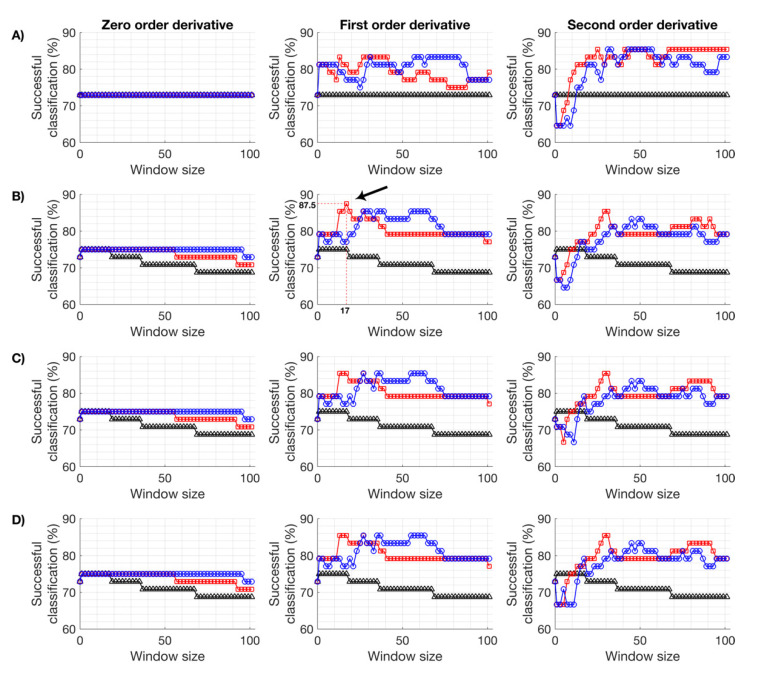
Effect of Savitzky–Golay (SG) and Loopy Multiplicative Scatter Correction (LMSC) parameters on the performance of Partial Least Squares Discriminant Analysis (PLS-DA) predictive models. For SG filtering, each derivative order (columns) was queried across a constant (black triangle), quadratic (red square), and quartic (blue circle) order polynomial, and each of these was then followed by zero, one, two, and three iterations of LMSC (rows **A**–**D**, respectively). The highest successful classification (%) of all combinations of SG and LMSC parameters was highlighted (arrow).

**Figure 2:**
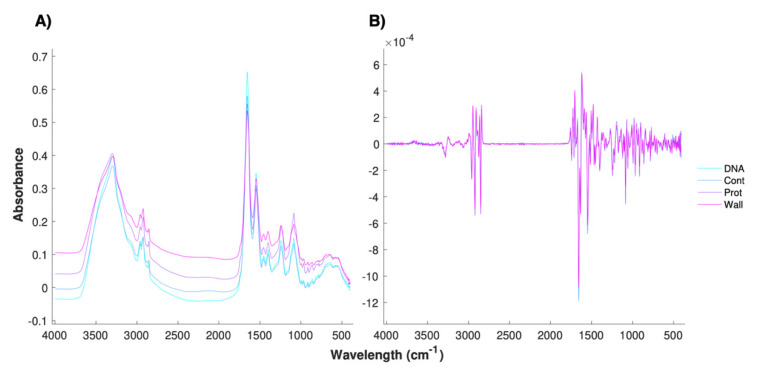
Average spectra of independent cultures exposed to Ciprofloxacin (DNA), Control (Cont), Kanamycin (Prot), and Amoxicillin (Wall) before any manipulation (**A**) and after the application of the optimal preprocessing strategy (**B**). These antibiotics were chosen as representatives of the major affected biosynthetic pathways.

FTIR spectra are composed of a sample-specific component, which ideally is closely related to the biological information of interest, and an unspecific and undesirable component, which is due to variability induced by environmental, experimental, and technical conditions. The objective of spectral pre-processing is to reduce the latter and highlight the biological relevance of the data [38]. Two commonly used preprocessing strategies are derivative filtering, typically with the Savitzky–Golay (SG) filter, followed by scattering correction, for instance the Loopy Multiplicative Scatter Correction (LMSC) algorithm [39]. Importantly, the performance of these preprocessing strategies depends on their parametrization, and this in turn differs with the system being studied [40]. To identify parameters that yield optimal predictive performance of the PLS-DA model, the successful classification after Leave-One-Out Cross-Validation (LOO-CV) was used (Figure 1). A single iteration of LMSC preceded by SG filtering with a window size of 17 datapoints, to which a quadratic polynomial was fitted to determine the first derivative, resulted in the optimal performance of a PLS-DA model, which is discussed over the following sections. The effect of the optimal preprocessing strategy on the raw spectra has been shown for three antibiotics acting on the major biosynthetic pathways (Figure 2). Additionally, the Extended Multiplicative Scatter Correction (EMSC) algorithm was explored but the performance of the PLS-DA did not improve substantially (data not shown). EMSC requires the intrinsic model of the dataset to be used to preprocess new spectra. Alternatively, MSC and LMSC are only dependent on a reference spectrum, which in this case was the average of mechanical replicas. Given the lack of performance improvement and the increased complexity of data analysis, the decision to not pursue EMSC as a preprocessing algorithm was taken.

### 2.3. Predicting the Major Biosynthetic Pathway Targeted

**Figure 3:**
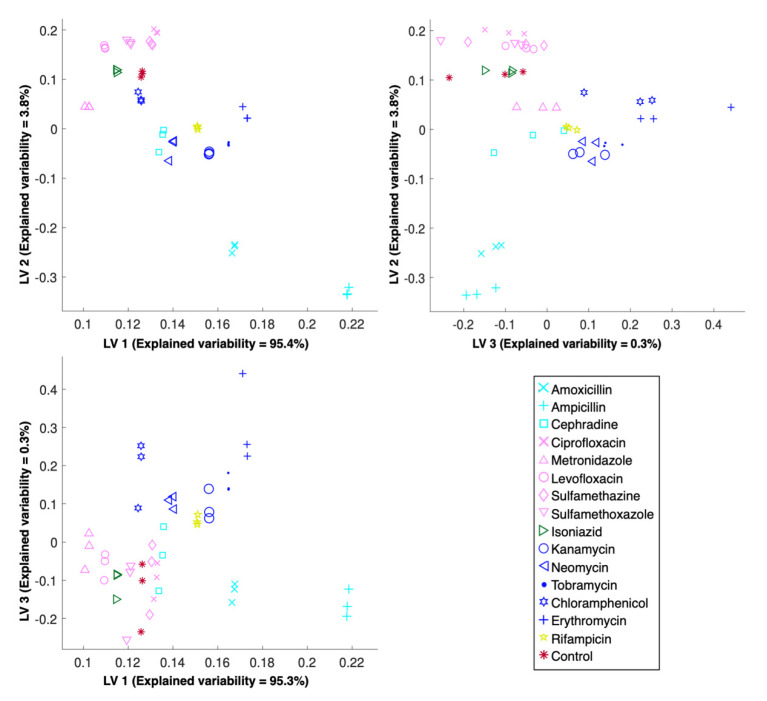
Representation of the metabolic fingerprints induced by antibiotics acting on the major biosynthetic pathways after Partial Least Squares Discriminant Analysis (PLS-DA), preceded by an optimized combination of preprocessing algorithms. The variability explained by each Latent Variable (LV) is reported for each corresponding axis. Antibiotics acting on the same major biosynthesis pathway are represented using the same color, and individual antibiotics are represented with a unique symbol.

**Figure 4:**
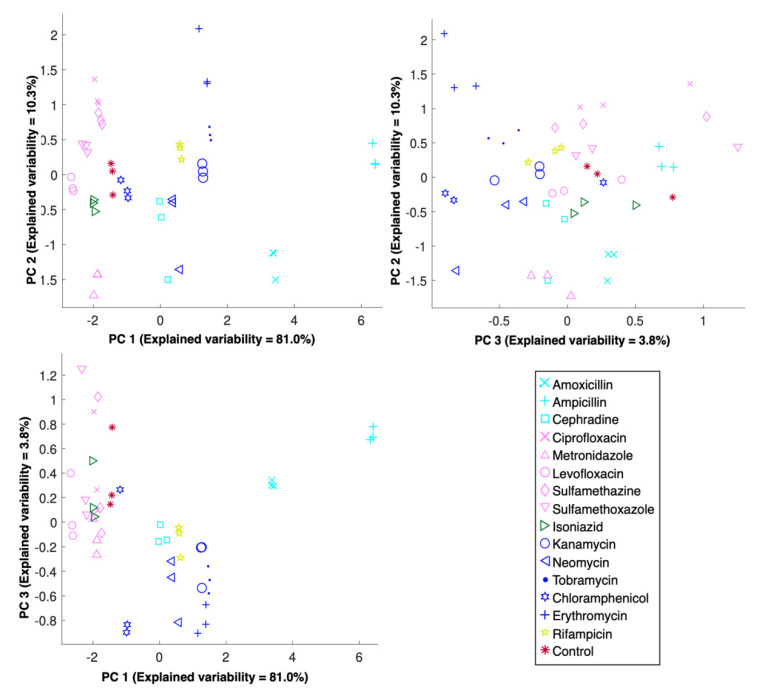
Representation of the metabolic fingerprints induced by 15 antibiotics acting on the major biosynthetic pathways after Principal Component Analysis (PCA), preceded by an optimized combination of preprocessing algorithms. The variability explained by each Principal Component (PC) is reported for each corresponding axis. Antibiotics acting on the same major biosynthesis pathway are represented using the same color, and individual antibiotics are represented with a unique symbol.

Pinpointing the MOA of a candidate molecule requires identifying its molecular target; however, this is a dauting task that must often be decomposed into smaller elements, the first of which is predicting the major biosynthetic pathway targeted. For that, a PLS-DA model was built with the optimized preprocessing parameters (Figure 3). Note that, with these parameters, a successful classification of 87.5% was obtained after LOO-CV (Figure 1). Importantly, the control samples, i.e., those exposed to the solvent but not the antibiotic, were predicted as different from all the other samples. This is particularly important as it allows one to differentiate the cases where no biosynthetic pathway was affected; therefore, the lack of drug effect can be predicted for candidate molecules that have no metabolic effect. Additionally, the metabolic fingerprints induced by antibiotics targeting each of the major biosynthetic pathways were separated with as little as two latent variables, as a very simple model was sufficient to explain over 99% of the spectral variability. To ensure that the observed clusters are indeed intrinsic to the spectra, since PLS-DA score plots can often be misleading and misinterpreted [41], a PCA was conducted on the same dataset, preceded by the exact same optimal preprocessing (Figure 4). The objective of this analysis was to reinforce the PLS-DA conclusions, rather than derive new ones. Unlike the predictive model built with PLS-DA, PCA is an unsupervised technique that directly reflects the inherent structure of the data. As such, a slightly higher intra-replica variability is observed, i.e., biological replicas are slightly more disperse. Ultimately, the similarity between the PLS-DA and PCA is a good indicator that the observed results are not the results of a fortunate combination of preprocessing and PLS-DA or an artifact of using the PLS algorithm for classification instead of calibration, for which it was originally implemented. As such, these results suggest that the observed results are a direct consequence of the capability of the proposed high-throughput FTIR spectroscopy screening assay in detecting metabolic fingerprints, particularly those induced by the exposure to different antibiotics acting on the major biosynthetic pathways.

### 2.4. Discriminating the Metabolic Fingerprints of Protein Synthesis Inhibitors

The next logical step in pinpointing the MOA of a candidate molecule is to discriminate between molecules that act with a similar MOA, e.g., on the same biosynthetic pathway but on a different point of the pathway. Within the clusters of metabolic fingerprints representing the major biosynthetic pathways targeted, there are sub-clusters coherent with the antibiotic classes tested (Figure 3 and Figure 4). For instance, for antibiotics that act on protein biosynthesis, those belonging to the aminoglycoside class (kanamycin, neomycin and tobramycin) have a metabolic fingerprint that is more similar among them than those belonging to the amphenicol (chloramphenicol) and the macrolide (erythromycin) classes. Interestingly, antibiotics of the aminoglycoside class bind to the 30S ribosomal unit, more specifically at the A-site, where they mimic the stabilization induced by cognate tRNA, thereby allowing noncognate tRNA to bind to the A-site, resulting in mRNA misreading and faulty protein synthesis. Additionally, allosteric binding sites affect ribosomal subunit mobility, which reduces translational activity and impairs ribosomal recycling. However, the specific relationship of these effects and cell death are not fully understood [42]. On the other hand, chloramphenicol, which belongs to the amphenicol class of antibiotics, has been considered a ‘general’ translation elongation inhibitor. Chloramphenicol was assumed to be a competitive inhibitor of aminoacyl-tRNA binding in the peptidyl transferase center of the 50S subunit A site, but recent studies suggest an MOA closer to that of macrolides, namely a sequence-specific inhibition of translation elongation [43]. Similarly, macrolides where thought to indiscriminately block protein elongation via a ‘plug-in-the-bottle’ mechanism, where binding to the tunnel close to the peptidyl transferase center physically obstructs nascent chain progression, but recent studies indicate that several proteins can bypass this blockage, thereby suggesting a sequence-specific mechanism [44]. Regardless of the specific MOA of each class, the antibiotics tested that target protein biosynthesis act at the elongation step; therefore, the fact that these were reproducibly plotted separately for three independent cultures suggests FTIR spectroscopy is not only capable of detecting metabolic fingerprints with sufficient sensitivity to elucidate MOA beyond targeting protein biosynthesis, but can conceivably be used to elucidate different mechanisms that disrupt protein elongation.

### 2.5. Discerning the Metabolic Fingerprints of DNA Synthesis Inhibitors

Regarding the antibiotics that inhibit DNA biosynthesis, the fluoroquinolones (levofloxacin and ciprofloxacin) were clustered together with the sulfonamides (sulfamethazine and sulfamethoxazole), but these were clearly distinct from metronidazole (Figure 3 and Figure 4). Fluoroquinolones block the progression of the enzyme–DNA complex formed during replication, which ultimately impairs DNA synthesis and induces rapid bacterial death. Specifically, fluoroquinolones MOA is based on the disruption of two enzymes: DNA gyrase, which introduces negative superhelical twists that facilitate the separation of daughter chromosomes and allows for the binding of initiation proteins; and topoisomerase IV, which is responsible for removing the interlinking of daughter chromosomes, therefore allowing their segregation into the daughter cells at the end of a replication round [45]. On the other hand, sulfonamides (sulfamethoxazole and sulfamethazine) are known as non-classical antifolates. This class of molecules are competitive inhibitors with *p*-aminobenzoic acid, preventing its entrance to the reaction site of dihydropteroate synthase and forming an analogue that cannot be used in the subsequent reactions, thereby greatly reducing folate levels. Because bacteria cannot absorb exogenous folate, thymine depletion occurs, and ultimately DNA biosynthesis errors, which result in the observed antibiotic effect [46]. Thymine depletion induces thymineless death, for which a consensual mechanism has not been proposed. One possible mechanism revolves around stalled replication forks [47], which, if confirmed, implies that the MOA of fluoroquinolones and sulfonamides could be more similar than traditionally acknowledged, which is in accordance with the obtained results. Alternatively, it could be that, despite having dissimilar MOAs, the metabolic fingerprint captured by FTIR spectroscopy is not sufficiently specific to distinguish between said MOAs. Lastly, although the MOA of metronidazole is still unclear, it is believed that metronidazole is intracellularly reduced to a short-lived nitroso free radical, which is not only cytotoxic, but also inhibits DNA synthesis and causes DNA damage by oxidation, which results in DNA degradation and eventually cell death [48]. This, in turn, is a considerably different MOA from both fluoroquinolones and sulfonamides, which is coherent with the results obtained. As a note, metronidazole is only intracellularly reduced in the presence of a sufficiently negative redox potential, and it could therefore be that the experimental setup utilized induced sufficient anaerobic conditions to obtain an antibiotic effect reflected on the metabolic fingerprint, since the facultative anaerobe model organism utilized, i.e., *E. coli*, can be susceptible to metronidazole [49] and apparently was (Table 1), but could also justify the proximity between samples exposed to metronidazole to the control samples. In sum, while the proposed FTIR spectroscopy bioassay is apparently not the most adequate tool to reach conclusions regarding the MOA of sulfonamides, it is possible that FTIR spectroscopy captures the metabolic fingerprint induced by antibiotics with sufficient sensitivity to distinguish those targeting DNA biosynthesis via different mechanisms.

### 2.6. Differentiating the Metabolic Fingerprints of Cell Wall Biosynthesis Inhibitors

Unlike the antibiotics described so far, those targeting cell wall biosynthesis had to be analyzed differently. Specifically, the standard concentration of four times the MIC resulted in extensive cell lysis, which in turn implied a considerable loss of intensity of the FTIR spectra. As such, cells were exposed to ampicillin at the MIC and to amoxicillin at 25% of the MIC. Cells were exposed to cephradine at the standard concentration, i.e., four times the MIC. Although this might explain the higher dispersion obtained for these antibiotics, in comparison with those targeting either DNA or protein biosynthesis, it was necessary to obtain spectra with a sufficient signal-to-noise ratio for analysis. Regardless of the distance between clusters of independent cultures exposed to the same antibiotic, there was coherence in the within-cluster distance, i.e., independent cultures were grouped together (Figure 3 and Figure 4), which is a positive indication. Briefly, amoxicillin, ampicillin, and cephradine are beta-lactam antibiotics and only differ in their affinities and/or molecular target. Beta-lactams inhibit transpeptidases and prevent cross-linking, thereby inducing structural deficiencies in the cell wall that results in cell lysis [50]. However, the mechanism of cell death induced by beta-lactams has been shown to extend beyond cell lysis. In fact, it seems that the cell wall synthesis machinery is recruited to a futile cycle of synthesis/degradation that depletes cellular resources and bolsters the bactericidal activity of beta-lactams [51]. Although the issue of the antibiotic concentration is a question that still lingers and must be attended for the industrial application of FTIR spectroscopy as a viable screening technology for antibiotic discovery, it is interesting to note that cephradine, which belongs to the cephalosporins sub-class of antibiotics, is clustered further away from the aminopenicillins (amoxicillin and ampicillin). If further validated, this could be another positive indication of the metabolic sensitivity of fingerprints obtained with FTIR spectroscopy.

### 2.7. Differentiating the Other Metabolic Fingerprints 

Interestingly, the samples exposed to isoniazid were clustered closely to those exposed to DNA synthesis inhibitors (Figure 3 and Figure 4). Isoniazid enters the cell as a pro-drug and exerts its antibiotic effect by disturbing various macromolecular syntheses, of which the most frequently discussed is mycolic acid synthesis; therefore, isoniazid is the preferred therapeutic for tuberculosis [52]. Although the MOA is still unclear, the peroxidative activation of isoniazid by the mycobacterial enzyme KatG forms potent inhibitors of lipid and nucleic acid biosynthesis, as well as inducing oxidative stress [53]. Regardless, its inhibitory effect on *E. coli* has long been reported as being dependent on the initial cell concentration, antibiotic concentration, and medium composition [54], and experimentally confirmed (Table 1). Apparently, the metabolic fingerprint detected with FTIR spectroscopy more closely reflects the inhibition of DNA biosynthesis; however, further validation of this observation is required. On the other hand, rifampicin samples were clustered together with protein synthesis inhibitors. Since rifampicin binds with high affinity to the bacterial DNA-dependent RNA polymerase, this results in its inhibition, ultimately causing a lethal disruption of RNA biosynthesis at the elongation step [55]. This suggests that the clustering observed is coherent given this type of inhibition. In other words, inhibition at the level of transcription should provide a metabolic effect that is closer to protein synthesis inhibition, which is at the level of translation, in comparison with DNA biosynthesis inhibition that occurs at the level of replication. Moreover, this is particularly distinct from cell wall biosynthesis inhibition, which is at a distant end of the spectrum of cellular metabolic responsibilities.

## 3. Conclusions

Given the importance of MOA identification in phenotypic screening, and the role of the latter for the success of antibiotic discovery, methods capable of combining MOA elucidation with high-throughput screening of whole cells are required now more than ever. Here, we explored FTIR spectroscopy as a metabolic fingerprinting technique regarding its sensitivity towards elucidating MOA, ranging from its looser definition of drug effects, e.g., the major biosynthetic pathway affected, through to the stricter drug target that individual antibiotics inhibit. Because FTIR spectroscopy requires extensive data analysis, a combination of parameters of commonly applied preprocessing algorithms was optimized. This ensured that the performance of predictive models was maximized. In general, the metabolic fingerprints obtained with FTIR spectroscopy were closely related to the MOA of all the antibiotics tested, which include examples that target protein, DNA, and cell wall biosynthesis. Additionally, the metabolic fingerprints induced by exposure to an RNA biosynthesis inhibitor was similar with those of protein synthesis inhibitors, which is coherent with the metabolic effect expected. Pending further validation, these fingerprints could help to elucidate the MOA of known drugs, for instance isoniazid, which was clustered close to DNA synthesis inhibitors, and also the thymineless death induced by sulfonamides. Ultimately, these results demonstrate that there is great potential in using FTIR spectroscopy as a tool to acquire a holistic picture of the effect of different antibiotics on the cellular metabolism, which can be used not only for antibiotic discovery but also towards a better understanding of the MOA of current antibiotics.

## 4. Materials and Methods

### 4.1. Antibiotic Stock Solutions and Susceptibility Testing

Antibiotic stock solutions of 15 compounds, belonging to 9 classes acting on 4 key biosynthetic pathways (Table 1), were prepared at a concentration of 4096 µg/mL, adjusted for potency, and kept at −20 °C or 4 °C, per recommendation. For in vitro susceptibility testing, the CLSI guidelines were followed [56] as well as the EUCAST documentation [57]. In detail, 100 µL of antibiotic solution was serially diluted in flatbottom 96-well plates, to which 100 µL of fresh cation-adjusted Mueller-Hinton broth (MHB) (VWR, Portugal) was added, along with 5 µL of cell suspension to obtain a concentration of 5 × 10^5^ Colony Forming Units per mL (CFU/mL). The bacteria were incubated at 37 °C for 24 h, after which growth inhibition was observed. MICs were determined as the lowest concentration at which no bacterial growth was observed for three independent cultures, and the inoculum size was confirmed by plating on cation-adjusted Mueller-Hinton Agar (MHA) and determining the CFU/mL.

### 4.2. Bacterial Cultures and Antibiotic Exposure

The bacterium E. coli strain JM 101 (ATCC33876) was chosen as a model organism for its ease of manipulation, non-pathogenic nature (biosafety class 1), as well as lack of resistance mechanisms in its genome: supE thi-1 Δ(lac-proAB) [F´ traD36 proAB lacIqZΔM15]. The bacteria were grown in1 L erlenmeyers, with 400 mL of MHB, in an orbital incubator (TH30 and SM30, Edmund Buhler GmbH) at 37 °C and 250 rpm. The cells were incubated until OD_590_ reached 0.270 ± 0.03, thereby ensuring cells were in the exponential growth phase. For exposure to each antibiotic, 18 mL of culture broth was transferred to a conical centrifugal tube with 7 mL of antibiotic stock solution to obtain a final concentration of four times the MIC. Cells were perturbed for 3-h in an orbital incubator at 250 rpm, 37 °C. The complete procedure was repeated for three independent cultures, conducted over different days. After antibiotic exposure, bacterial inactivation was confirmed by counting CFU/mL, as described elsewhere [58].

### 4.3. Spectral Data Acquisition, Preprocessing, and Multivariate Analysis

After incubation with the antibiotics, samples were quickly centrifuged at 3000 Relative Centrifugal Force (RCF) for 10 min at 4 °C (Rotanta 460R, Hettich Zentrifugen, Germany), the supernatant was discarded, and the cell pellet was resuspended in 25 mL of cold 0.9% NaCl (Merck, Germany) to quench the metabolism. Subsequently, the OD_590_ was taken and bacterial inactivation was determined. The cells were, again, pelleted and resuspended in cold 0.9% NaCl to obtain an OD_590_ of 1, from which 2 mL of aliquots were further centrifuged for three minutes at 13,000 rpm (13,793 g) (Z160M, Hermle Labortechnik, Germany) and resuspended in 100 µL of cold 0.9% NaCl to obtain an OD_590_ of 20 for FTIR readings. The samples were then plated on an infrared-transparent ZnSe 96 well plate (Bruker, Germany) in quintuplicates. ZnSe plates were dehydrated for 3 h in a vacuum desiccator with silica, and inserted in a HTS-XT module coupled to a Vertex-70 spectrometer (Bruker Optics, Germany). Spectra were acquired in transmission mode and consisted of 40 coadded scans at a 4-cm^−1^ resolution. These were then exported as data point table files, which were imported into Matlab (Matworks, USA) for subsequent analysis. To reduce spectra heterogeneity originating from operator handling and other undesirable sources of variability, the spectra of mechanical replicates (quintuplicates) were averaged. Subsequently, the averaged spectra were preprocessed with the SG filter and then with LMSC. A range of parameters were used for either algorithm, and the parameters that produced the highest successful classification of an LOO-CV PLS-DA model were used to build the final PLS-DA predictive model as well as for PCA. 

## Figures and Tables

**Table 1:** Classification of the antibiotics tested, the biosynthetic pathway targeted and their Minimum Inhibitory Concentration (MIC). The average bacterial inactivation after exposure to 4 X MIC for 3 h was determined by plate counting Colony Forming Units (CFUs). The results are shown as the percentage variation in regard to the control, which confirms the desired cell death.

Antibiotic	MIC (µg/mL)	Average Inactivation (%)	Class	Biosynthetic Pathway Targeted
***Amoxicillin***	8	99.8	Beta-lactam	Cell Wall
***Ampicillin***	8	100	Beta-lactam	Cell Wall
***Cephradine***	8	99.7	Beta-lactam	Cell Wall
***Chloramphenicol***	4	94.3	Amphenicol	Protein
***Ciprofloxacin***	0.5	100	Fluoroquinolone	DNA
***Erythromycin***	32	93.2	Macrolide	Protein
***Isoniazid***	256	93	Other	Other
***Kanamycin***	8	100	Aminoglycoside	Protein
***Levofloxacin***	0.125	100	Fluoroquinolone	DNA
***Metronidazole***	128	96.3	Nitroimidazole	DNA
***Neomycin***	2	100	Aminoglycoside	Protein
***Rifampicin***	32	100	Rifamycin	RNA
***Sulfamethazine***	8	99.8	Sulfonamide	DNA
***Sulfamethoxazole***	32	98.9	Sulfonamide	DNA
***Tobramycin***	2	100	Aminoglycoside	Protein
